# CHANGES IN TIBIALIS ANTERIOR MUSCLE ACTIVITY FOLLOWING TIBIAL NERVE BLOCK IN ADULTS WITH SPASTIC EQUINOVARUS FOOT: AN OBSERVATIONAL PILOT STUDY

**DOI:** 10.2340/jrm.v57.44405

**Published:** 2025-12-01

**Authors:** Alessandro PICELLI, Mirko FILIPPETTI, Angelo PONTILLO, Eleonora DIMITROVA, Nicola VALÈ, Rita DI CENSO, Nicola SMANIA, Matteo BERTUCCO

**Affiliations:** 1 Department of Neurosciences, Biomedicine and Movement Sciences, University of Verona, Verona; 2 Neurorehabilitation Unit, University Hospital of Verona, Verona, Italy; 3 Canadian Advances in Neuro-Orthopaedics for Spasticity Consortium (CANOSC), Kingston, ON, Canada; 4 Department of Engineering for Innovation Medicine, University of Verona, Verona, Italy

**Keywords:** electromyography, muscle spasticity, rehabilitation, symptom assessment

## Abstract

**Objective:**

To investigate changes in tibialis anterior and gastrocnemius medialis activity during walking following a diagnostic nerve block of the tibial nerve in patients with spastic equinovarus foot.

**Design:**

Observational pilot study.

**Patients:**

Thirteen adult patients (10 males, 3 females) with spastic equinovarus foot due to stroke or traumatic brain injury.

**Methods:**

All patients underwent a diagnostic nerve block of the tibial nerve main trunk. Clinical assessments included passive and active ankle range of motion, spasticity grading using the Modified Ashworth Scale and the Tardieu Scale, and muscle strength evaluation. Surface electromyography was recorded during gait to analyse muscle activation and coactivation patterns before and after the procedure.

**Results:**

Passive ankle dorsiflexion significantly improved after the nerve block. Spasticity scores decreased, while active range of motion and Tardieu angle (passive range of motion during slow stretch – angle at which resistance is first felt during fastest stretch) showed trends toward improvement. Electromyographic analysis revealed reduced activation of both tibialis anterior and gastrocnemius medialis, along with a significant decrease in coactivation.

**Conclusion:**

Diagnostic nerve block of the tibial nerve effectively reduces spasticity and alters antagonist muscle activity, supporting its role in personalized assessment and treatment planning for spastic equinovarus foot.

## LAY ABSTRACT

People who have had a stroke or brain injury often develop a condition where their foot turns inward and points down, making walking difficult. This is called spastic equinovarus foot. In this study, we tested a simple and safe procedure that temporarily blocks a nerve in the leg to see how it affects walking. We looked at how 2 key leg muscles worked before and after the procedure using sensors placed on the skin. We found that the treatment helped reduce muscle tightness and improved ankle movement. Interestingly, it also changed how the muscles worked together during walking. These results suggest that this nerve block can help doctors better understand the cause of walking problems and choose the most effective treatment for each patient. This approach could lead to more personalized and successful rehabilitation strategies.

Spastic equinovarus foot (SEVF) is one of the most prevalent patterns of lower limb spasticity following stroke or acquired brain injury (1). It is characterized by plantarflexion and inversion of the foot, which can significantly impair gait, balance, and overall mobility, thereby reducing independence and quality of life (1).

SEVF is not a uniform condition; rather, it results from a complex interplay of neuromuscular impairments that vary across individuals. The main contributors to SEVF include overactivity of the calf muscles (gastrocnemius, soleus, and tibialis posterior), muscle–tendon shortening, weakness of ankle dorsiflexors, and imbalanced activation between the tibialis anterior (TA) and peroneal muscles (2). These factors often coexist, and their relative contribution may differ from patient to patient. As a result, patients with similar clinical presentations may have different underlying pathophysiological mechanisms (3). This variability complicates the clinical assessment and makes it difficult to select the most appropriate treatment based solely on physical examination (4).

To address this challenge, diagnostic nerve block (DNB) has emerged as a valuable tool (2). DNB involves the injection of a local anaesthetic to temporarily block nerve conduction, allowing clinicians to assess the role of spasticity in a given motor pattern (5). When applied to the tibial nerve and its motor branches, DNB can help determine whether spastic overactivity of the calf muscles is a primary contributor to SEVF (2). A positive response (such as reduced muscle tone, improved passive range of motion, and better foot alignment) suggests that spasticity is a key factor (2, 6, 7). This information is essential for guiding interventions such as botulinum toxin injections, phenol neurolysis, or surgical procedures (2).

In addition to clinical observation, surface electromyography (sEMG) during gait analysis has proven valuable in identifying abnormal muscle activation patterns (3). sEMG can reveal out-of-phase activation of the plantar flexors during the swing phase, which is indicative of spastic overactivity. Conversely, minimal or absent continuous activity may suggest a fixed contracture of the plantar flexors or weakness of the dorsiflexors (3).

It is important to note that coactivation of agonist and antagonist muscles at the ankle is not exclusively pathological. During normal gait, a certain degree of coactivation contributes to joint stiffness regulation and postural stability (8). In hemiparetic subjects, increased coactivation may represent a compensatory strategy to enhance stability, particularly in the presence of impaired balance or muscle weakness, although excessive coactivation can reduce gait efficiency and increase energy cost (8).

Given the reciprocal relationship between agonist and antagonist muscles, it is plausible that reducing spastic overactivity in the calf muscles through tibial nerve DNB may influence the activity of ankle dorsiflexors, particularly the TA. However, to date, there is still a lack of research specifically addressing this interaction. The aim of this observational pilot study was to explore changes in TA muscle activity following tibial nerve DNB in adults with SEVF due to stroke or acquired brain injury.

## METHODS

This observational pilot study was conducted at our rehabilitation unit between March and July 2022. The study protocol was approved by the Review Board of our Department, and all participants provided informed consent prior to enrolment. The study was conducted in accordance with the ethical principles outlined in the Declaration of Helsinki.

### Participants

Participants were recruited based on the following inclusion criteria: age >18 years; diagnosis of SEVF secondary to a first-ever stroke or acquired brain injury, confirmed by computed tomography or magnetic resonance imaging; time since onset ≥ 6 months; spasticity of the calf muscles graded ≥ 2 on the Modified Ashworth Scale (MAS) (9); no prior treatment with botulinum toxin type A for calf muscle spasticity; and ability to walk independently for at least 10 m barefoot. Exclusion criteria included: participation in other clinical trials; fixed contractures (MAS = 4) or bony deformities of the affected limb; previous neurolytic or surgical treatment for SEVF; anticoagulant therapy; and other neurological or orthopaedic conditions affecting the lower limb.

### Tibial nerve diagnostic block

A DNB of the tibial nerve main trunk was performed under ultrasound guidance (MyLab 70 XVision, Esaote, Italy) and electrical nerve stimulation (Plexygon, Vygon, Italy). The tibial nerve was identified by eliciting a motor response to a 1 Hz, 100 μs, 0.5 mA stimulus. After negative aspiration to avoid intravascular injection, 6 mL of 2% lidocaine was administered using a 22-gauge, 50 mm echogenic needle (SonoPlex STIM, Pajunk, Germany) (10). The effectiveness of the block was confirmed by the abolition of the Achilles tendon reflex, assessed by the same physician who performed the procedure.

### Clinical assessment

Clinical and demographic data were collected at baseline (T0) and immediately after the DNB (T1). Passive and active range of motion (PROM and AROM) for ankle dorsiflexion and plantarflexion were assessed using a handheld goniometer. Measurements were taken with the patient in a supine position and the knee fully extended (6, 7). Spasticity was assessed using the MAS and the Tardieu Scale (grade and angle) (9, 11). The MAS is a 6-point scale grading the resistance of a relaxed limb to rapid passive stretch (0 = no increase in muscle tone; 1 = slight increase in muscle tone at the end of the range of motion; 1+ = slight increase in muscle tone through less than half of the range of motion; 2 = more marked increase in muscle tone through most of the range of motion; 3 = considerable increase in muscle tone; 4 = joint is rigid) (11). The Tardieu Scale was used to grade the muscle’s response to the fastest passive stretch in ankle dorsiflexion. It categorizes the reaction from 0 to 4: 0 indicates no resistance throughout the movement; 1 reflects slight resistance; 2 denotes a clear catch at a specific angle with interruption followed by release; 3 indicates unsustained clonus at a precise angle; and 4 represents sustained clonus at a precise angle. In addition to grading, the scale quantifies spasticity through the spasticity angle, calculated as the difference between R2 (the passive range of motion during slow stretch) and R1 (the angle at which resistance is first felt during fast stretch) (9). For statistical purposes, MAS scores were rescaled (e.g., 1+ = 2, 2 = 3, etc.). The angle of paresis (PROM–AROM) was calculated for ankle dorsiflexion Furthermore, strength of the ankle dorsiflexors was evaluated using the Medical Research Council (MRC) scale (0–5) (12).

### Instrumental assessment

sEMG was recorded using a wireless system (BTS FREEEMG 300, BTS, Italy) at a sampling rate of 1000 Hz. Electrodes were placed over the TA and gastrocnemius medialis (GM) muscles of the affected limb according to SENIAM guidelines (13). Patients walked barefoot along a 10-m walkway at a self-selected speed (gait speed was not standardized between pre- and post-block assessments). Two identical sEMG sessions were performed at T0 and T1. A G-WALK inertial sensor (BTS, Italy) was positioned at the lumbosacral junction to identify gait phases, with data sampled at 100 Hz.

sEMG outcomes were summarized as the area under the rectified, filtered, and time‑normalized curve over the full gait cycle. Phase‑specific (stance/swing) and timing (onset/offset) analyses were not performed in this pilot. For the sake of clarity, the term “area of activation” refers to the area under the curve of the rectified and filtered sEMG signal for each gait cycle, normalized to the subject’s maximum activation.

This measure reflects the overall amplitude and duration of muscle activity during walking, similar to integrated EMG in classical analyses. The “coactivation coefficient” was calculated as the overlapping area between the normalized activation curves of the tibialis anterior and gastrocnemius medialis within the same gait cycle. This index quantifies the degree of simultaneous activation of these antagonist muscles. In the Results section, “reduction in activity” indicates a decrease in the area of activation, meaning lower overall muscle activation during gait. Thus, there is a direct relationship between “area of activation” and “activity” in our terminology.

### Data analysis

EMG signals were processed using custom MATLAB scripts (MathWorks, Natick, MA, USA). Signals were bandpass filtered (5–450 Hz), full-wave rectified, notch filtered at 50 Hz, and low-pass filtered at 100 Hz using a 5th-order Butterworth filter (14). The area under the curve of the TA and GM signals was calculated for each gait cycle, time-normalized, and averaged. Amplitude normalization was performed using each subject’s maximum activation. The coactivation index was computed as the overlapping area between normalized TA and GM activation curves within the same gait cycle. This approach is consistent with previous studies on muscle coactivation during gait (8). Although formal test–retest reliability was not assessed in this pilot study, the method is based on established normalization and overlap computation procedures. No frequency-domain analysis was performed. Descriptive statistics included medians and interquartile ranges (IQR). Pre-post comparisons were analysed using the Wilcoxon signed-rank test. Statistical analyses were performed using SPSS 26.0 (IBM, USA) and Stata/IC 15.1 (StataCorp, College Station, TX, USA).

## RESULTS

### Participants

Thirteen patients were included in the study: 12 with chronic stroke and 1 with traumatic brain injury. The sample consisted of 10 males and 3 females. The median age was 64 years (IQR = 51; 70), and the median time since injury was 5 years (IQR = 4; 11). All participants completed the intervention and assessments. No adverse events or complications related to the DNB were reported, confirming the safety of the procedure.

### Clinical outcomes

Following the DNB, a statistically significant improvement was observed in the PROM of ankle dorsiflexion (*p* < 0.001), indicating improved joint mobility. A trend toward significance was also noted for AROM (*p* = 0.066). Spasticity measures showed significant reductions post-treatment. MAS scores decreased significantly (*p* = 0.002), reflecting a reduction in muscle tone. Similarly, the Tardieu spasticity grade showed a significant decrease (*p* = 0.005), indicating a reduction in the velocity-dependent component of spasticity. A trend toward significance was also noted for the Tardieu angle of spasticity (*p* = 0.063). A significant improvement (*p* = 0.009) was also found for the angle of paresis. No significant changes were observed in the strength of ankle dorsiflexors, as expected, because the DNB targets the tibial nerve and does not directly affect dorsiflexor innervation. A summary of clinical outcomes is presented in Table I.

**Table I:** Clinical outcomes of study sample.

Outcome	Before DNBMedian (IQR)	After DNBMedian (IQR)	Comparison after DNB vs before DNB*p*-value (Z)
Ankle PROM (degrees)	0 (–10; 0)	5 (0; 10)	0.001* (–3.241)
Ankle AROM (degrees)	–10 (–20; –10)	–10 (–20; –10)	0.066 (–1.841)
MAS (0–5)	4 (3; 4)	2 (1; 3)	0.002* (–3.169)
Tardieu Scale – grade (0–4)	2 (1; 2)	1 (1; 1)	0.005* (–2.810)
Tardieu Scale – angle of spasticity (degrees)	10 (5; 10)	5 (5; 10)	0.063 (–1.857)
Angle of paresis (degrees)	10 (7.5; 10)	15 (12.5; 20)	0.009* (–2.612)
MRC ankle dorsiflexors (0–5)	3 (1; 4)	3 (1; 4)	0.18 (–1.342)

IQR: Interquartile range; PROM: passive range of motion; AROM: active range of motion; MRC: Medical Research Council; MAS: Modified Ashworth Scale.

* Statistically significant (*p*< 0.05).

### Instrumental outcomes

**Fig. 1:**
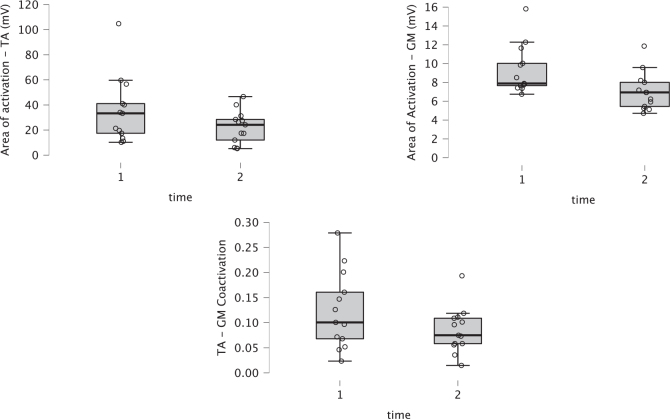
Boxplot of instrumental outcome measures pre- and post-treatment. Top left: area of activation of the tibialis anterior (TA) muscle. Top right: area of activation of the gastrocnemius medialis (GM) muscle. Bottom: TA–GM coactivation.

**Fig. 2:**
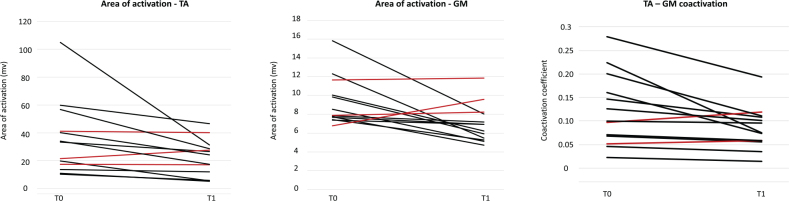
Surface electromyography: single-subject results pre- and post-treatment. Left: area of activation of tibialis anterior (TA) muscle. Middle: area of activation of the gastrocnemius medialis (GM) muscle. Right: TA–GM coactivation. Red lines indicate increased post-treatment values.

sEMG analysis revealed significant changes in muscle activity patterns. The area of activation for both GM and TA muscles significantly decreased after the DNB (*p* = 0.01 and *p* = 0.006, respectively). The reduction in GM activity reflects the expected effect of the nerve block on the spastic plantar flexors. Interestingly, the decrease in TA activity suggests a modulation of antagonist muscle behaviour, possibly due to reduced co-contraction or improved motor efficiency. The coactivation coefficient between TA and GM also significantly decreased post-treatment (*p* = 0.01). The reported reductions in TA activity refer to whole‑cycle area‑of‑activation measures and should not be interpreted as phase‑specific changes. See Figs 1 and 2 for more details.

## DISCUSSION

In this observational pilot study, we examined the effects of a tibial nerve DNB on both clinical and electrophysiological parameters in patients with SEVF secondary to stroke or acquired brain injury. Specifically, we analysed changes in TA and GM activity during gait, as measured by sEMG, following administration of a DNB at the main trunk of the tibial nerve. Our findings provide novel insights into the interaction between spastic plantar flexors and their antagonists and support the use of DNB combined with sEMG as complementary tools for the clinical assessment and management of SEVF.

From a clinical standpoint, the DNB produced significant improvements in ankle PROM, as well as reductions in spasticity measured by the MAS and the Tardieu grade. These findings are consistent with previous work showing that tibial nerve blocks reduce calf muscle overactivity and improve joint mobility in patients with SEVF (6, 7). The observed trend toward improvement in AROM and in the Tardieu spasticity angle in our cohort further supports the potential functional benefits of this intervention, although these changes did not reach statistical significance in this pilot study. In line with a retrospective study by Lamora and colleagues, which reported significant improvements in active ankle dorsiflexion and reductions in both spasticity and paresis angles after tibial nerve block and selective tibial neurotomy (15), our dataset showed an exploratory decrease in the paresis angle (ankle dorsiflexion PROM–AROM) after the block. This observation is compatible with reduced co‑contractions and improved voluntary control and suggests that incorporating the paresis angle as a predefined outcome could add value in future studies.

The instrumental analysis revealed a significant reduction in the area of activation for both GM and TA during walking after the DNB, accompanied by a reduction in TA–GM coactivation. The decrease in GM activity was expected due to the direct effect of the anaesthetic on tibial-innervated plantar flexors. By contrast, the reduction in TA activity was unexpected given its established role in swing‑phase dorsiflexion. A plausible interpretation is that, prior to the block, TA activation was exaggerated to overcome plantar‑flexor overactivity; once that resistance was suppressed, TA activation normalized, yielding lower amplitude but potentially more efficient recruitment. Importantly, we did not perform gait kinematics; therefore, we cannot confirm whether equinus during swing improved or whether swing‑phase dorsiflexion increased. Nevertheless, the reduction in coactivation together with the trend towards improved AROM suggests more selective TA recruitment. These points underscore that sEMG amplitude alone does not fully capture functional improvement; reduced amplitude may reflect improved coordination rather than weakness. These findings are consistent with principles of motor control and reciprocal inhibition, whereby antagonist activity is modulated in response to changes in agonist tone (16). Temporarily removing spastic input through DNB can help restore a more physiological activation pattern, including in muscles not directly targeted by the block.

An alternative explanation for the TA reduction is unintended diffusion of the anaesthetic toward the common peroneal nerve; although ultrasound guidance and technique aimed to minimize this risk, minimal spread cannot be entirely excluded. Future work using nerve‑specific stimulation or imaging‑based diffusion tracking could clarify this possibility.

Inter‑individual variability provided further insights. In a minority of cases, GM activity did not decrease after DNB. In such cases, the most likely explanation is an incomplete nerve block. While structural contributors (e.g., muscle–tendon shortening) can sustain an equinus deformity, they do not explain persistent EMG activity, which reflects neural overactivity (7). Conversely, a reduction in GM activity with persistent TA activation may reflect compensatory recruitment or incomplete recovery of dorsiflexor control. In cases where GM activity remained unchanged after the block, the main contributor to the deformity may be passive (7). Although PROM and MAS improved in all patients, these measures alone cannot distinguish between spasticity and structural changes. The Tardieu Scale, which includes both a qualitative grade and a spasticity angle, is designed to address this distinction. In our cohort, the Tardieu grade decreased significantly after DNB, suggesting a neural component; however, the spasticity angle showed only a trend towards improvement.

TA activation during swing is a normal physiological pattern; its persistence may represent an ongoing strategy to ensure foot clearance in the presence of residual motor impairment rather than a “dysfunction”. Based on these observations, we propose a pragmatic clinical reasoning framework for interpreting DNB and sEMG findings in SEVF. If the DNB does not reduce the equinus pattern/deformity, muscle shortening should be considered a primary determinant, and interventions such as stretching, casting, or surgery may be more appropriate. If the DNB reduces the equinus pattern and reveals improved dorsiflexor function, targeted interventions such as botulinum toxin type A injections may be indicated to sustain the benefits of the block (2, 6, 7). In this context, the observed reduction in TA sEMG after the block should not be construed as impaired dorsiflexor function; rather, it may signal normalization of activation once excessive compensatory recruitment is no longer required. Consequently, treatment decisions should prioritize functional measures (e.g., active dorsiflexion performance and gait kinematics) over sEMG amplitude alone.

An apparent contradiction merits explicit consideration: TA sEMG decreased after the block, whereas dorsiflexor weakness is frequently observed clinically in SEVF, particularly in equinus during swing. One might argue that reducing TA activation could worsen foot clearance. Our interpretation is that lower TA amplitude most likely reflects normalization and improved efficiency in the setting of reduced antagonist resistance, not a deterioration of dorsiflexor performance. However, without phase‑specific sEMG and kinematic data (e.g., ankle dorsiflexion during swing and minimum toe clearance), we cannot determine whether swing‑phase dorsiflexion improved or worsened. Future studies should therefore integrate phase‑resolved sEMG (stance vs swing), onset/offset timing, and gait kinematics and examine their relationships with clinical metrics, including the paresis angle.

Finally, our results underline both the added value and practical constraints of sEMG in clinical settings. While clinical scales (PROM, MAS, Tardieu) improved across patients, these measures alone cannot disentangle neural overactivity from structural changes. sEMG contributes complementary information on activation patterns and coactivation that can refine phenotyping and guide personalized treatment (3). At the same time, barriers to widespread sEMG adoption (technical complexity, time, and training) remain relevant in routine practice (17). Even so, basic yet robust metrics – such as integrated activity (area of activation) and a transparent coactivation index – can yield clinically meaningful insights in pilot and pragmatic studies.

The observed reduction in TA activity after tibial nerve block raises important considerations for rehabilitation. While this decrease likely reflects normalization rather than weakness, clinicians should monitor dorsiflexor performance to ensure adequate foot clearance during gait. If residual weakness or insufficient dorsiflexion persists, targeted interventions such as strengthening exercises or functional electrical stimulation may be warranted to optimize motor recovery and prevent compensatory strategies. Regarding existing literature, most published work focuses on plantar flexor muscles and clinical outcomes rather than antagonist muscle activation patterns in patients with SEVF. Specifically, we note that evidence on TA sEMG after botulinum toxin injection into the calf muscles or ankle–foot orthosis use is scarce. This gap highlights the novelty of our work.

This study has several limitations. First, the small sample size limits the generalizability of our findings and reduces statistical power to detect subtle effects. Second, the sEMG analysis was restricted to time-normalized activation areas without examining the temporal dynamics of muscle activation. Future studies should include detailed timing analyses, such as onset and offset of muscle activity, to better characterize gait patterns. Third, the short-term nature of the assessment did not allow evaluation of the duration of DNB effects or their functional impact on gait performance. Fourth, we did not collect gait kinematic data, which prevents us from determining whether changes in TA sEMG corresponded to actual improvements in SEVF during walking. This limitation is critical because reduced TA activation could reflect normalization rather than impaired dorsiflexion; without kinematic measures (e.g., ankle dorsiflexion during swing or toe clearance), this interpretation remains speculative. Fifth, gait speed was self-selected and not standardized between pre- and post-block assessments, which may have influenced EMG amplitude and coactivation measures. Sixth, we did not compute phase-resolved TA activity (stance vs swing) or onset/offset timing, which would have provided additional insight into functional changes. Seventh, the reliability of the coactivation index was not formally tested, and no healthy control group was included for comparison, limiting interpretation of whether observed coactivation values differ from physiological patterns. It is also noteworthy that the median Tardieu grade before the block was 2, which may appear lower than expected given the frequent presence of clonus in the triceps surae. This finding likely reflects our limited sample size, assessment position (knee extended), and possible adaptations due to chronicity or prior rehabilitation. Finally, we did not measure ankle plantar-flexor strength. This decision was based on the expected effect of the tibial nerve block on these muscles, which was considered clinically evident, and on the study’s primary focus on antagonist muscle activity during gait. Future research should include plantar-flexor strength assessment and integrate kinematic and phase-specific sEMG analyses to provide a more comprehensive understanding of neuromuscular and functional changes.

Nevertheless, this study provides preliminary evidence that tibial nerve DNB can influence not only the targeted spastic muscles but also their antagonists, potentially improving overall motor control. To our knowledge, this is among the first studies to examine the effects of tibial nerve DNB on TA activity during gait in patients with SEVF. These findings may inform clinical decision-making and support a more individualized approach to spasticity management.

In conclusion, integrating DNB with sEMG analysis offers a promising strategy for the assessment and treatment planning of SEVF in patients with acquired brain injury. By identifying the specific contributions of spasticity, muscle shortening, and motor control deficits, clinicians can better tailor interventions to each patient’s unique presentation. Further research with larger cohorts and longitudinal follow-up is warranted to confirm these findings and clarify their implications for rehabilitation outcomes.
